# Effect of Shikonin Against *Candida albicans* Biofilms

**DOI:** 10.3389/fmicb.2019.01085

**Published:** 2019-05-14

**Authors:** Yu Yan, Fei Tan, Hao Miao, Hui Wang, YingYing Cao

**Affiliations:** ^1^Shanghai Skin Disease Hospital, Tongji University School of Medicine, Shanghai, China; ^2^Institute of Basic Medicine, Chengde Medical University, Chengde, China; ^3^School of Pharmacy, Second Military Medical University, Shanghai, China

**Keywords:** *Candida albicans*, biofilms, shikonin, farnesol, hyphae

## Abstract

Candidiasis is often associated with the formation of biofilms. *Candida albicans* biofilms are inherently resistant to many clinical antifungal agents and have increasingly been found to be the sources of *C. albicans* infections. Novel antifungal agents against *C. albicans* biofilms are urgently needed. The aim of this study was to investigate the effect of shikonin (SK) against *C. albicans* biofilms and to clarify the underlying mechanisms. XTT reduction assay showed that SK could not only inhibit the formation of biofilms but also destroy the maintenance of mature biofilms. In a mouse vulvovaginal candidiasis (VVC) model, the fungal burden was remarkably reduced upon SK treatment. Further study showed that SK could inhibit hyphae formation and reduce cellular surface hydrophobicity (CSH). Real-time reverse transcription-PCR analysis revealed that several hypha- and adhesion-specific genes were differentially expressed in SK-treated biofilm, including the downregulation of ECE1, HWP1, EFG1, CPH1, RAS1, ALS1, ALS3, CSH1 and upregulation of TUP1, NRG1, BCR1. Moreover, SK induced the production of farnesol, a quorum sensing molecule, and exogenous addition of farnesol enhanced the antibiofilm activity of SK. Taken together, these results indicated that SK could be a favorable antifungal agent in the clinical management of *C. albicans* biofilms.

## Introduction

*Candida albicans* is a pleiomorphic fungal pathogen of humans which may cause superficial to life-threatening infections (Pfaller and Diekema, 2007; Azie et al., 2012). The predisposing factors for *C. albicans* infections include antibiotic therapy, immunosuppressive therapy, human immunodeficiency virus (HIV) infection, diabetes and old age. In addition, structured microbial communities attached to medical devices or human organs, commonly referred to as biofilms, have increasingly been found to be the sources of *C. albicans* infections (Donlan and Costerton, 2002; Gulati and Nobile, 2016). The exist of *C. albicans* biofilm can exacerbate clinical infections through forming a reservoir for producing recalcitrant pathogenic cells, which act as seeds to disseminate the organism to bloodstream and lead to invasive systemic infection. Moreover, *C. albicans* biofilms show unique phenotypic traits, the most outstanding of which is that they are notoriously resistant to a wide variety of clinical antifungal agents, including fluconazole and conventional amphotericin B (Chandra et al., 2001; Tobudic et al., 2010; Nett et al., 2011). Therefore, there is an urgent need to develop new antifungal agents against *C. albicans* biofilms.

Shikonin (SK) is the major constituent of the red pigment extracts from the roots of the plant *Lithospermum erythrorhizon*. SK is widely used as a material to prepare an ointment to treat burn, wounds, and hemorrhoids (Vassilios et al., 1999; Sasaki et al., 2002). Besides, SK shows possible therapeutic roles in brain disorders and gastric cancers (Nam et al., 2008; Lee et al., 2014). It has been reported that SK exhibits extensive pharmacological activities, including antiviral, antitumor, antidiabetic, anti-inflammatory, and antithrombotic effects (Chen et al., 2002; Sasaki et al., 2002; Lu et al., 2011; Lan et al., 2014). In our previous studies, we demonstrated the effect of SK against planktonic *C. albicans* cells, including the azole-resistant clinical isolates (Miao et al., 2012; Liao et al., 2016). However, the role of SK in *C. albicans* biofilms has not yet been investigated. In this study, we investigated the activity of SK against *C. albicans* biofilms and explored the underlying mechanisms.

## Materials and Methods

### Strains, Media, and Compounds

*C. albicans* strain SC5314 was obtained from professor Dominique Sanglard (Centre Hospitalier Universitaire Vaudois, Lausanne, Switzerland). All clinical *C. albicans* isolates are obtained from Changhai Hospital of Shanghai, China. *C. albicans* cells were routinely maintained on Sabouraud dextrose agar (1% w/v peptone, 4% w/v dextrose, and 1.8% w/v agar) and grown in YPD liquid medium (1% yeast extract, 2% peptone, and 2% dextrose) at in an orbital shaker at 30°C (Zhu et al., 2013). For all the experiments *in vitro*, 6.4 mg/ml SK (purity > 98%, National Institutes for Food and Drug Control, Peking, China) in dimethyl sulfoxide and 25 mM farnesol (Sigma-Aldrich, United States) in methanol were used as stock solutions and added to the culture suspensions to obtain the required concentrations. Spider and Lee’s media used for hyphae formation were made as previously described (Gimeno et al., 1992; Liu et al., 1994). RPMI-1640 medium (Gibco, United States) was used for biofilm formation experiments.

### Antifungal Susceptibility Test

Antifungal susceptibility testing was performed in 96-well tissue culture plates (Corning Inc., NY, United States) using a broth microdilution protocol of the Clinical and Laboratory Standards Institute M27-A3 method, with a few modifications (Quan et al., 2006). In brief, the initial concentration of fungal cells in RPMI-1640 medium was 5 × 10^3^ CFU/ml, and the final concentrations ranged from 0.125 to 64 μg/ml for SK and fluconazole. Plates were incubated at 35°C for 24 h. The MIC_80_ was determined as the lowest concentration of the drugs that inhibited growth by 80%.

### *In vitro* Biofilm Formation Assay

The *in vitro* biofilm formation assay was carried out as described previously (Ramage et al., 2001). In brief, *C. albicans* cells (1.0 × 10^6^ cells/ml) in RPMI-1640 medium were added to a 96-well tissue culture plate (Corning Inc., Corning, NY, United States) for 90 min of adhesion at 37°C. For RNA extraction, cells were introduced into a 75 cm^2^ tissue culture flask (Thermo Fisher Scientific Inc., Waltham, MA, United States). Following the initial 90 min of adhesion, the medium was aspirated and non-adherent cells were removed and then fresh medium was added to the adherent cells. The plate was further incubated at 37°C for 24 h until formation of mature biofilms. To test the effect of the compounds (SK, farnesol) on *C. albicans* biofilm formation, different concentrations of the compounds were added to fresh RPMI-1640 after 90 min of adhesion. To detect the effect of SK on mature *C. albicans* biofilms, biofilms were formed at 37°C for 24 h as described above. The biofilms were washed with PBS, then fresh RPMI-1640 medium containing different concentrations of SK was added. The plates were incubated at 37°C for a further 24 h to detect the antibiofilm effect of SK.

### XTT Reduction Assay

The growth of biofilms was measured with a 2,3-bis-(2-methoxy-4-nitro-5- sulfophenyl)-2H-tetrazolium-5-carboxanilide (XTT) reduction assay, a reaction catalyzed by mitochondrial dehydrogenases (Ramage et al., 2001). In brief, biofilm cells were washed with PBS and then incubated with 0.5 mg/ml of XTT and 1 mM of menadione in PBS at 37°C for 90 min. The optical density (OD) was determined at 490 nm using a microtiter plate reader. The SMIC_80_ was determined as the lowest concentration of the drugs that inhibited mature biofilm by 80%.

### Biofilm Biomass Measurement

The measurement of biofilm biomass was conducted as described previously (Nobile et al., 2006) with slight modifications. Silicone disks (1.5 × 1.5 cm, Bentec Medical Corp., United States) were weighed before inoculation. *C. albicans* cells (1.0 × 10^6^ cells/ml) in RPMI-1640 medium were added to a 12-well tissue culture plate with one prepared silicone disk in each well. The inoculated plate was incubated with for 90 min at 37°C. Then the medium was aspirated, non-adherent cells were removed and fresh medium was added to the adherent cells. The plate was further incubated gentle agitation at 37°C for 24 h until the formation of mature biofilms. To test the effect of SK on biomass production, different concentrations of SK were added to fresh RPMI-1640 after 90 min of adhesion. The silicone disks with attached biofilms were removed from the wells, dried overnight and weighed. The total biomass of biofilm was calculated by subtracting the weight of the silicone prior to biofilm growth from the weight of the silicone after the drying period, with adjustment for the weight of control silicone squares exposed to no cells.

### Hyphae Formation

Three hypha-inducing media (Lee’s medium, Spider medium and YPD medium supplemented with 10% fetal bovine serum) was used to test the effect of SK on hyphal formation. *C. albicans* (5.0 × 10^5^ cells/ml) were suspended in the hypha-inducing media containing 0.5 μg/ml of SK and added to 24-well tissue culture plates. The plates were incubated at 37°C for 3 h. The morphology of the cells was photographed under microscope.

### Confocal Laser Scanning Microscopy (CLSM)

To observe the effect of SK on *C. albicans* biofilms formation with CLSM, siliconedisks (Bentec Medical Corp., United States) were used to develop biofilms. Biofilms were washed and incubated for 45 min at 37°C in PBS containing the fluorescent stains FUN-1 (10 μM) (Molecular Probes, Eugene, OR, United States) and concanavalin A-Alexa Fluor488 conjugate (ConA) (25 μg/ml) (Molecular Probes, Eugene, OR, United States). FUN-1 is converted to an orange-red cylindrical intravacuolar structure by metabolically active cells, whilst ConA binds to the glucose and mannose residues of cell wall polysaccharides and emits a green fluorescence. After incubation with the dyes, the disks were flipped and the stained biofilms were observed with a Leica TCS SP2 confocal laser scanning microscope equipped with argon and HeNe lasers.

### Farnesol Measurement

Farnesol was extracted and measured as previously described (Hornby et al., 2001) with some modificaton. In brief, the supernatant of *C. albicans* biofilm treated with SK for 24 h was collected. The biofilm cells were scraped from the culture plate and filtered through filters (0.45 μm) and washed three times with ultrapure sterilized water. Cells were dried at room temperature to constant weight and weighed using an electronic balance. Farnesol in the supernatants was extracted using ethyl acetate and detected with HPLC (Agilent, United States). The detection was performed by absorbance at 210 nm. A 5 mm C18 reversed-phase column (4.6 by 250 mm) was utilized with a mobile phase of 4:1 methanol-H_2_O eluted at a flow rate of 1 ml/min. Six different concentrations of farnesol standard samples were used to create a standard curve of integral areas eluting at its retention time. The extracted farnesol was calculated based on the HPLC integral area compared with a standard curve. Farnesol concentrations obtained from each culture were also normalized on the biofilm dry weight assays.

### Cellular Surface Hydrophobicity(CSH) Assay

The cellular surface hydrophobicity (CSH) of *C. albicans* biofilms was determined by a water-hydrocarbon two-phase assay as described previously (Klotz et al., 1985). In brief, the formed *C. albicans* biofilms were removed from the culture plate surface with a sterile scraper to obtain a cell suspension (OD_600_ = 1.0 in YPD medium). A total of 1.2 ml of the suspension was pipetted into a clean glass tube and overlaid with 0.3 ml of octane. The mixture was vortexed for 3 min and then stood at room temperature for phase separation. Soon after the two phases had separated, the OD_600_ of the aqueous phase was determined, and the OD_600_for the group without the octane overlay was used as the control. Relative CSH was calculated as follows: [(OD_600_ of the control-OD_600_ after octane overlay)/OD_600_of the control] × 100.

### Real-Time RT-PCR

RNA isolation and real-time RT-PCR were performed as described previously (Li et al., 2014). Total RNA was isolated with fungal RNAout kit (TIANZ, Beijing, China) according to the manufacturer’s protocol. The isolated RNA was resuspended in diethyl pyrocarbonate-treated water. The OD was measured at 260 nm and 280 nm. The integrity of the RNA was visualized by subjecting 2–5 μl of the samples to electrophoresis through a 1% agarose-MOPS gel. The cDNA was obtained using the cDNA Synthesis Kit for RT-PCR (TaKaRa Biotechnology, Dalian, China). Real-time PCR was performed with a 7500 real-time PCR system (Applied Biosystems). SYBR green I (TaKaRa Biotechnology, Dalian, China) was used to monitor the amplified products in real time according to the manufacturer’s protocol. The primers are shown in Table 1. The PCR protocol consisted of denaturation program (95°C for 10 s), 40 cycles of amplification and quantification program (95°C for 10 s, 60°C for 20 s, 72°C for 15 s with a single fluorescence measurement), melting curve program (60–95°C with a heating rate of 0.1°C per second and a continuous fluorescence measurement) and finally a cooling step to 40°C. The expression of each gene was normalized to that of 18S rRNA. The relative expression of each target gene was calibrated against the corresponding expression in untreated biofilms.

**Table 1:** Primers used in this study.

Primer	Sequence
ECE1-F^a^	GCTGGTATCATTGCTGATAT
ECE1-R	TTCGATGGATTGTTGAACAC
HWP1-F	TGGTGCTATTACTATTCCGG
HWP1-R	CAATAATAGCAGCACCGAAG
EFG1-F	TATGCCCCAGCAAACAACTG
EFG1-R	TTGTTGTCCTGCTGTCTGTC
CPH1-F	ATGCAACACTATTTATACCTC
CPH1-R	ATGCAACACTATTTATACCTC
RAS1-F	GGCCATGAGAGAACAATATA
RAS1-R	GTCTTTCCATTTCTAAATCAC
ALS1-F	TTGGGTTGGTCCTTAGATGG
ALS1-R	ATGATTCAAAGCGTCGTTC
ALS3-F	CTAATGCTGCTACGTATAATT
ALS3-R	CCTGAAATTGACATGTAGCA
CSH1-F	CTGTCGGTACTATGAGATTG
CSH1-R	GATGAATAAACCCAACAACT
TUP1-F	GATTGACGAG TCCTCCAACG
TUP1-R	AAACCAACCTATCGCCATCA
NRG1-F	TATCAGTATG CTGCTCCTCC
NRG1-R	GGAGTTGGCCAGTAAATCAC
BCR1-F	AGTATAATGCTCCTGGTAAGAA
BCR1-R	ACGTAAAGGAGGCACGGCATA
DPP3-F	TTATCTGTAATTATCATTGT
DPP3-R	GTTGTCAAACTTCAATTGA
18S rRNA-F	AATTACCCAATCCCGACAC
18S rRNA-R	TGCAACAACTTTAATATACGC


*^*a*^F, forward primer; R, reverse primer.*

### Evaluation of *in vivo* Antifungal Activity

A mouse vaginal model was used to demonstrate the antifungal effect of SK *in vivo* according to the previous procedures with slight modification (Yano and Fidel, 2011). Briefly, 3 days prior to inoculation, female KM clean mice (Shanghai SLAC Laboratory Animal Center, China) were injected with 0.1 mg of β-estradiol (Sigma-Aldrich, Shanghai, China) subcutaneously in the lower abdomen. Estrogens injections were administered every other day for three times in a week. The intravaginal inoculation with 4 × 10^6^ *C. albicans* cells in 20 μl of PBS was injected into vaginal lumen of the estrogen-treated mice. After the establishment of murine model, SK (dissovled in sesame oil) was delivered intravaginally on days 1, 2, 3, and 4 after inoculation. Sesame oil was employed as a negative control. On the fifth day, the vaginal lumens of the mice were lavaged by 100 μl of PBS with repeated aspiration and agitation with a pipette tip. The lavage fluids were picked and cultured on the YPD plates. The CFU was counted to analyzed the infection burdens. The animal experiments were approved by the Animal Ethics Committee of the Second Military Medical University (Shanghai, China).

### Statistical Criteria

Statistics were calculated in GraphPad Prism 6.0 (GraphPad Software, San Diego, CA, United States), in which *P*-value of < 0.05 or < 0.01 was considered statistically significant.

## Results

### SK Inhibits *C. albicans* Biofilms

**FIGURE 1:**
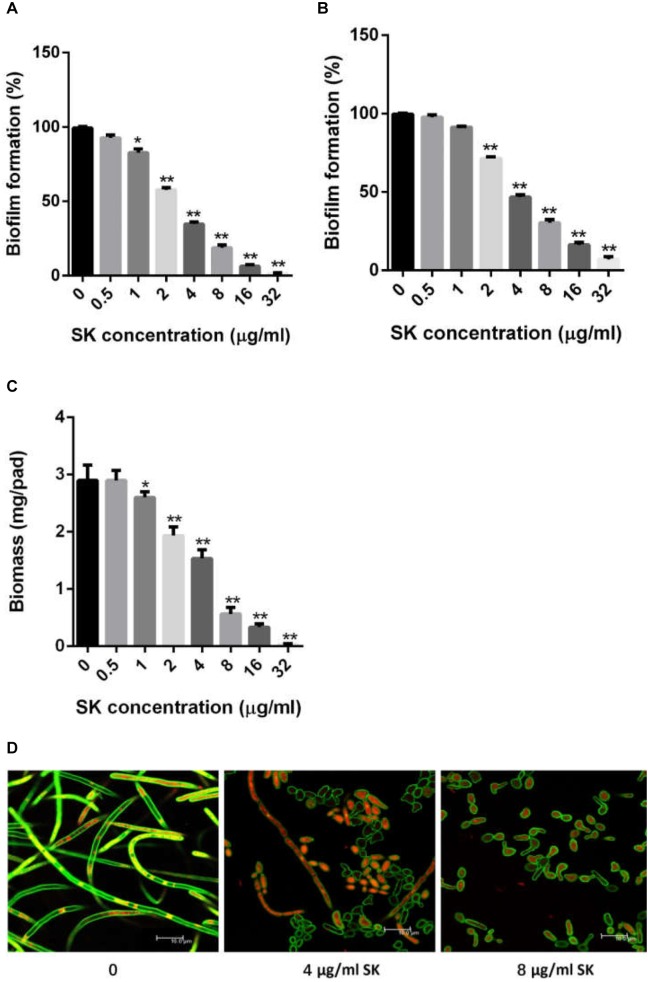
SK inhibits *C. albicans* biofilms *in vitro*. **(A)** Effects of different concentrations of SK on biofilm formation. **(B)** Effects of different concentrations of SK on mature biofilms. **(C)** Effects of different concentrations of SK on biofilms formation determined by biomass production. **(D)** Effects of different concentrations of SK on biofilm formation, shown in CLSM images. Data are expressed as the mean ± standard deviation of the independent assays in triplicate. ^∗^*P* < 0.05; ^∗∗^*P* < 0.01 as compared to the SK-free group.

We first determined the MIC (minimum inhibitory concentration) values of SK on *C. albicans* strains, including a standard strain SC5314 and several clinical isolates. As shown in Table 2, SK could inhibit both planktonic and biofilm cells. The MIC_80s_ of SK against plantonic *C. albicans* cells ranged from 2 to 4 μg/ml. It should be noted that SK exhibited similar antifungal activity on the fluconazole-resistant strains as the fluconazole-sensitive strains. The SMIC_80s_ of SK against *C. albicans* mature biofilms ranged from 4 to 16 μg/ml while the SMIC_80s_ of fluconazole were over 64 μg/ml. Figure 1A showed that SK inhibited biofilm formation in a dose-dependent manner when this compound was added to *C. albicans* cells after 90 min of adhesion. More specifically, 1 μg/ml of SK inhibited biofilm formation by 17.3%, and the inhibitory effect on biofilm formation was enhanced when the concentration of SK was increased. 4 μg/ml of SK inhibited biofilm formation by 65.4%, while the biofilm growth was almost totally inhibited upon exposure to 32 μg/ml of SK. The effect of SK against mature *C. albicans* biofilms was shown in Figure 1B. Although the mature biofilm cells were hardly affected at the SK concentrations of less than 1 μg/ml, almost half of the mature biofilm was destroyed when SK was added at the concentration of 4 μg/ml. SK at the concentration of 32 μg/ml destroyed mature biofilms by 92.8%.

**Table 2:** Effects of shikonin and fluconazole against *Candida albicans*.

*C. albicans* Strains	MIC_80_ for plantonic cells (μg/ml)^a^		SMIC_80_ for biofilm (μg/ml)^b^	
		
	SK	Fluconazole	SK	Fluconzole
SC5314	4	0.5	16	>64
8376	2	0.5	8	>64
6355	4	1	16	>64
4390	4	0.5	16	>64
6375	2	0.25	8	>64
5473	2	16	4	>64
6885	4	>64	16	>64
2336	4	>64	8	>64
9664	4	>64	16	>64
4647	2	>64	16	>64


*^*a*^MIC_*80*_ is the minimum inhibitory concentration of drug that inhibited planktonic cell growth by 80% compared with that of the drug-free cells. ^*b*^SMIC_*80*_ is minimum inhibitory concentration of drug that inhibited mature biofilm by 80% compared with that of the drug-free cells determined by XTT reduction assay.*

Consistent with the results of the XTT reduction assay, biofilm biomass determination revealed that SK could inhibit biomass production (Figure 1C). Addition of 1 μg/ml of SK led to a significant drop of biomass level as compared to the control group without SK treatment. The inhibitory effect on biomass production was enhanced when the concentration of SK was increased, while almost no biomass was produced in the presence of 32 μg/ml of SK.

Confocal laser scanning microscopy (CLSM) analysis further confirmed the antibiofilm activity of SK. In the SK-free group, *C. albicans* biofilm exhibited atypical three-dimensional nature, composed mainly of true hyphae. However, the structure of *C. albicans* biofilm was destroyed in the presence of SK. At the concentration of 4 μg/ml of SK, the biofilm was predominantly composed of yeast cells and pseudohyphae, while true hyphae were rarely observed. When 8 μg/ml of SK was added, the *C. albicans* biofilm displayed a poor architecture, with a complete disappear of true hyphae or pseudohyphae (Figure 1D).

### SK Inhibits *C. albicans* Hyphae Formation

**FIGURE 2:**
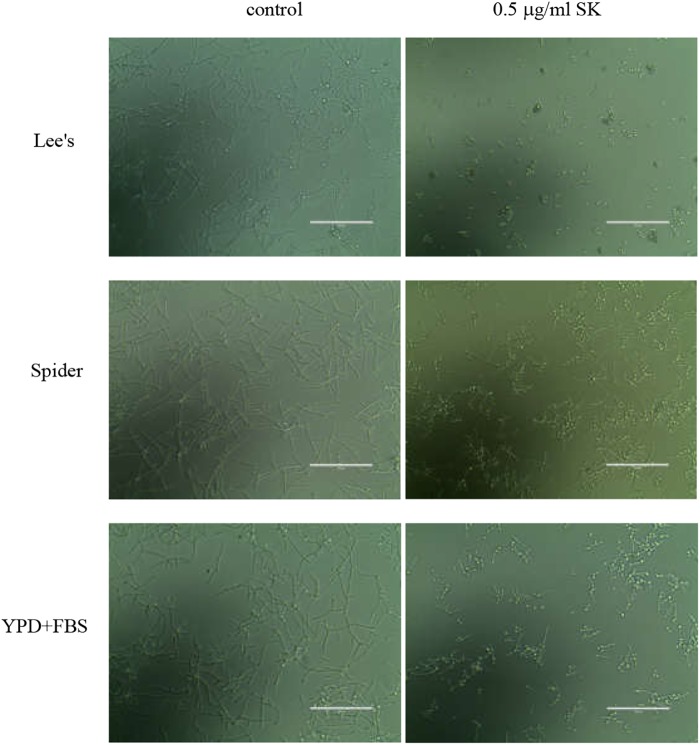
Effects of SK on hyphae formation. *C. albicans* SC5314 cells were incubated in Lee’s, Spider or YPD liquid media supplemented by 10% fetal bovine serum (YPD + FBS). The cellular morphology was photographed after incubation at 37°C for 3 h.

Since yeast-to-hypha growth is a key factor in *C. albicans* biofilm formation and virulence, we tested the effect of SK on filamentous growth. *C. albicans* cells were cultured in various hypha-inducing media, including Lee’s, Spider and YPD liquid media containing 10% fetal bovine serum. As shown in Figure 2, in the absence of SK, *C. albicans* underwent hyphal initiation, produced elongated and regular hyphal cells. When 0.5 μg/ml SK was added, the filamentous growth was remarkably inhibited. Specifically, the filamentation in Lee’s media was completely inhibited by 0.5 μg/ml of SK as only yeast cells were observed.

### SK Reduces Cellular Surface Hydrophobicity of *C. albicans* Biofilm

**FIGURE 3:**
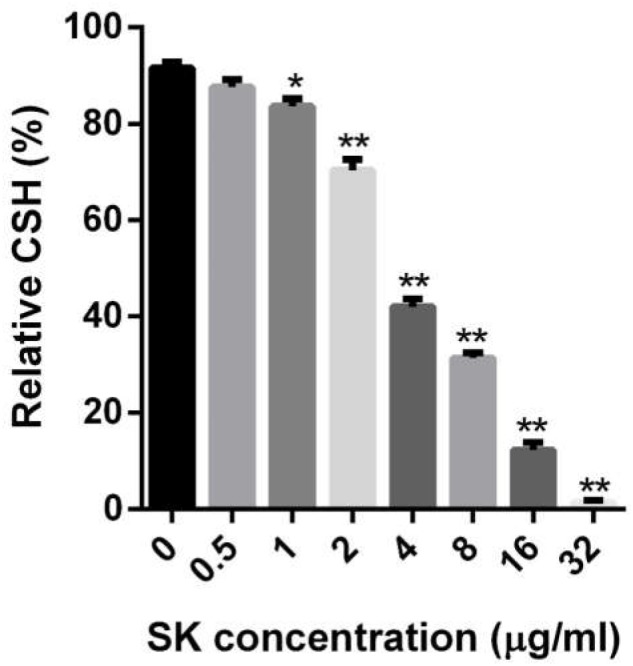
Effects of different concentrations of SK on the cell surface hydrophobicity (CSH) of *C. albicans* biofilms. CSH was estimated by the water-hydrocarbon two-phase assay. Data are expressed as the mean ± standard deviation of the independent assays in triplicate.^∗^*P* < 0.05; ^∗∗^*P* < 0.01 as compared to the SK-free group.

The CSH is an important factor for cell adhesion and biofilm formation, so we determined the CSH of *C. albicans* biofilms upon SK treatment. As shown in Figure 3, a negative correlation was observed between SK concentrations and the CSH of *C. albicans* biofilms. The relative CSH of untreated *C. albicans* biofilm was high (about 91.7%). SK decreased the CSH of biofilms in a dose-dependent manner. When the biofilm was treated with 2 μg/ml of SK, the relative CSH was significantly reduced, to approximately 70.3%. The relative CSH of the biofilm almost reached zero following the treatment of 32 μg/ml of SK.

### SK Regulates the Hypha- and Adhesion-Specific Genes Expression

**FIGURE 4:**
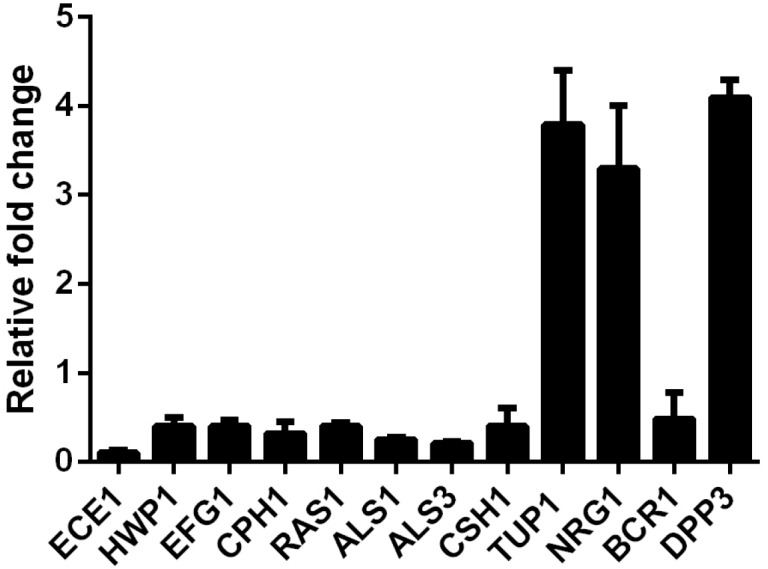
Transcription levels of several *C. albicans* genes following treatment with 2 μg/ml of SK were detected by real-time reverse transcription PCR (RT-PCR). The mRNA levels were normalized on the basis of their 18S rRNA levels. Gene expression was indicated as the fold increase in SK-treated biofilm cells relative to that of the untreated biofilm cells. Data are expressed as the mean ± standard deviation of the independent assays in triplicate.

In view of the inhibition of SK on *C. albicans* filamentous growth and adhesion, we investigated the expression of several well-known genes involved in hyphae formation and adhesion. In this experiment, 2 μg/ml of SK was used to inhibit biofilm formation for 8 h. The real-time reverse transcription-PCR (RT-PCR) results showed that ECE1, HWP1, EFG1, CPH1, RAS1, ALS1, ALS3, and CSH1 were downregulated. The upregulated genes included TUP1, NRG1, and BCR1 (Figure 4).

### SK Induces Farnesol Production

**FIGURE 5:**
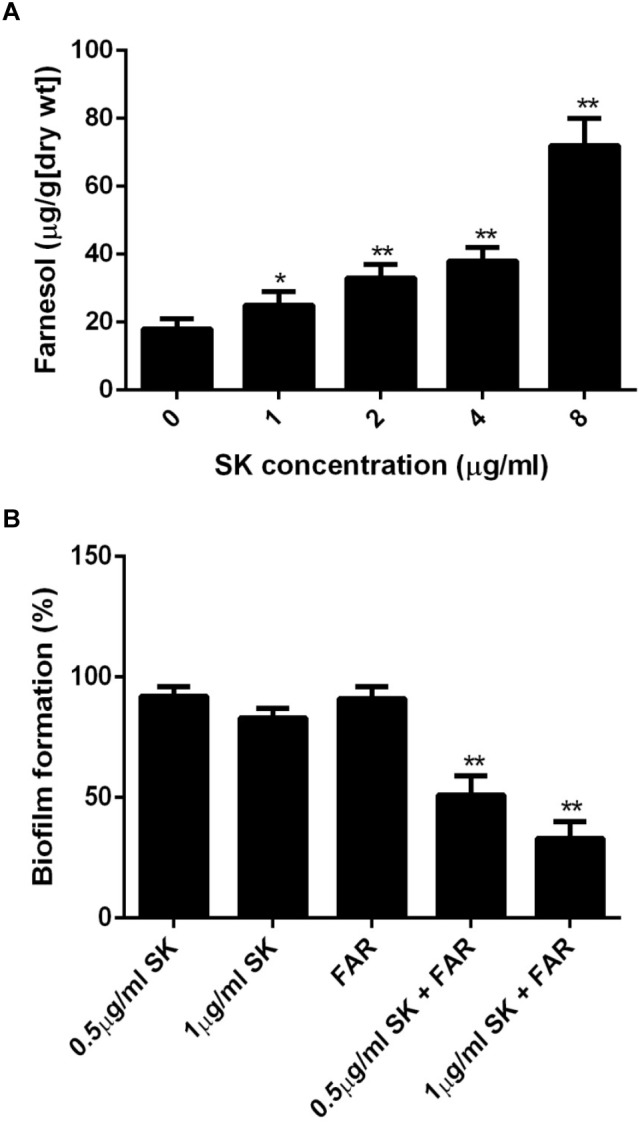
**(A)** Farnesol production in the supernatant of *C. albicans* biofilms treated with different concentrations of SK for 24 h. **(B)** Effect of farnesol on the antibiofilm activity of SK. *C. albicans* cells were cultured in biofilm formation condition. After 90 min of adhesion, SK (0.5, 1 μg/ml) or farnesol (25 μM) or the combination of the two compounds were added and the biofilm growth was continued for 24 h. Biofilm formation was evaluated using an XTT reduction assay, and the results are presented as the percent of compound-treated biofilms relative to the control (compound-free) biofilm. The results represent means ± standard deviations for three independent experiments. ^∗∗^*P* < 0.01 as compared to the corresponding SK-treated alone group.

It has been reported that farnesol, one of the quorum sensing molecules in *C. albicans*, played an important role in yeast-to-hypha transition and biofilm formation (Ramage et al., 2002). In view of the inhibition of hyphae and biofilm formation upon SK treatment, we hypothesized that SK might exert this effect through modulating the level of farnesol. As expected, our results revealed that SK increased the production of farnesol in a dose-dependent manner. The level of farnesol was four times higher in 8 μg/ml of SK-treated biofilm than that in SK-free biofilm (Figure 5A). Consistent with this, DPP3, which encodes a phosphatase and has been demonstrated to be responsible for farnesol synthesis by converting farnesyl pyrophosphate to farnesol (Navarathna et al., 2007), was remarkably upregulated upon SK treatment (Figure 4).

### Farnesol Enhances the Antibiofilm Activity of SK

Since the level of farnesol was increased upon SK treatment, we investigated the effect of farnesol on the antibiofilm activity of SK. To this end, the concentrations of SK (0.5 and 1 μg/ml) that had only a slight impact on biofilm formation were used. Similarly, 25 μM of farnesol used alone had no significant effect on biofilm. When SK was used in combination with farnesol, the formation of biofilms was significantly inhibited. More specifically, the combined treatment of 1 μg/ml of SK and 25 μM of farnesol resulted in approximately 40% biofilm formation rate as compared to drug-free biofilm (Figure 5B).

### *In vivo* Activity of SK on *C. albicans*

**FIGURE 6:**
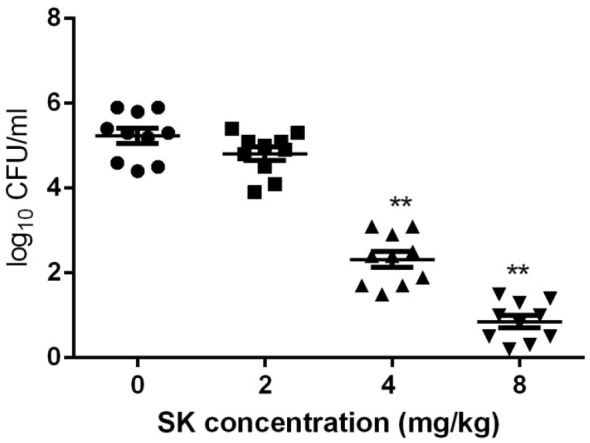
Fungal burden analyses by colony counting after the administrations of SK in a mouse vaginal model. Statistical significance among the groups was analyzed by one-way ANOVA. ^∗∗^*P* < 0.01 as compared to the values from the SK-free group.

Since *C. albicans* is believed to be commonly occurred in vulvovaginal candidiasis (VVC) and VVC infections mainly rely on biofilm formation (Sobel et al., 1998; Fazly et al., 2013), here the *in vivo* activity of SK was investigated using a mouse vaginal infection model. As shown in Figure 6, although treatment of 2 mg/kg of SK had a slight impact on the fungal burden, 4 mg/kg of SK-treated group showed approximately 3 log10 CFU/ml reduction of fungal burden as compared to the control (SK-free) group. There was an even remarkable drop of fungal burden when SK was administrated at the dose of 8 mg/kg, while the fungal cells seemed to be wiped out.

## Discussion

An important reason for the failure of current antifungal therapy is attributed to the formation of biofilms which are inherently resistant to most antifungal treatments. Thus, there is an urgent need for new antifungal drugs that can efficiently inhibit biofilms (Donlan and Costerton, 2002). In this study, a strong inhibitory effect of SK against *C. albicans* biofilms was revealed. More importantly, SK could not only inhibit the formation of *C. albicans* biofilms but also destroyed the maintenance of mature biofilms. The antifungal activity of SK was further confirmed in a mouse model of VVC. The mechanisms for the antibiofilm activity of SK included the inhibition of hyphae formation and adhesion as well as enhanced farnesol production.

The antifungal agents used in the treatment of *C. albicans* infections mainly include azoles (e.g., fluconazole and itraconaole), polyenes (e.g., amphotericin B) and echinocandins (e.g., caspofungin and micafungin). Although treatment of *C. albicans* biofilm was shown to be effective with polyenes and echinocandins in several reports, high drug resistance occurs in biofilms of *C. albicans* when treated with azoles, especially fluconazole which is widely used in clinical practice due to its relatively low cost and side effect (Anderson, 2005; Mukherjee et al., 2009; Kucharicová et al., 2013). It was reported that fluconazole could hardly affect mature *C. albicans* biofilm (Chandra et al., 2001). In this study, the antifungal effect of SK was outstanding as compared to fluconazole. SK could effectively inhibit *C. albicans* biofilm and 32 μg/ml of SK could destroy over 90% of mature biofilms, while the SMIC_80_ of fluconazole was over 64 μg/ml.

Vulvovaginal candidiasis (VVC) is the second most common infection disease in female genitourinary tract, and *C. albicans* is considered as the most common pathogen in this disease (Achkar and Fries, 2010; Spence, 2010). Recent studies reveal that VVC infections mainly rely on the formation of *C. albicans* biofilm and have been commonly used for *in vivo* evaluation of antibiofilm compounds (Gao et al., 2016; Liu et al., 2018). To evaluate the antibiofilm activity of SK *in vivo*, we chose a mouse VVC model. We found that SK could significantly alleviate vaginal fungal burden at the dose of 4 mg/kg. Moreover, when SK was administrated at the dose of 8 mg/kg, the fungal cells seemed to be wiped out. Collectively, our findings indicated that SK might exhibit great potential as a novel antibiofilm compound.

In recent years, cell-cell signaling, particularly quorum sensing, has been one of the focuses of microbiological research. *C. albicans* can produce farnesol as an extracellular quorum-sensing molecule, which is also the first quorum-sensing molecule discovered in a eukaryote (Shirtliff et al., 2009; Polke et al., 2018). It has been proved that farnesol inhibits *C. albicans* biofilm formation (Hornby et al., 2001; Kebaara et al., 2008). Here we found that SK could enhance farnesol production in *C. albicans* biofilms. Consistently, the expression of DPP3, the key gene involved in farnesol synthesis, was remarkably upregulated upon SK treatment. Previous studies demonstrated that farnesol exerted a synergistic or additive interaction with antifungal agents, including micafungin, fluconazole and amphotericin B, against *C. albicans* biofilms (Cordeiro et al., 2013). Similarly, our study showed that the combination of SK and farnesol resulted in a much higher antibiofilm activity than SK used alone. These results indicated that farnesol might play an important role in the antibiofilm mechanisms of SK.

In *C. albicans*, inhibition of yeast-to-hypha transition is a key role for farnesol. In fact, hyphal morphogenesis is required to establish robust biofilm with complex three-dimensional structure, and interruption of the yeast-to-hypha transition would prevent biofilm development. In this study, the structure of biofilm was destroyed by SK in a dose-dependent manner, and treatment of 8 μg/ml SK resulted in a poor biofilm architecture, with a complete disappear of true hyphae or pseudohyphae. The inhibition of filamentous growth by SK was also confirmed in hypha-inducing liquid media. These results were consistent with the inhibitory activity of farnesol on *C. albicans* morphological transition.

To uncover the underlying molecular basis of inhibited morphological transition and cell adhesion by SK, we assessed the transcription levels of genes related to hyphal growth and adhesion. Our real-time RT-PCR analysis revealed that several important hypha- and adhesion-specific genes were differentially expressed upon SK treatment. HWP1 is uniquely expressed on the hyphal surface, and biofilms lacking HWP1 were prone to detach from the abiotic substrate (Chaffin, 2008). RAS1 belongs to the Ras1-cAMP-protein kinase A (PKA) signaling pathway, a key regulator of the yeast-to-hypha switch and biofilm formation (Davis-Hanna et al., 2008). EFG1 and CPH1 are major transcription regulators of filamentous growth controlling many signaling pathways involved in filamentation (Nobile et al., 2012; Araújo et al., 2017). BCR1 is a transcriptional factor that regulates the adhesive properties of hyphal cells by affecting the expression of cell wall and adhesin genes, including members of the ALS family and HWP1 (Nobile and Mitchell, 2005; Nobile et al., 2006). The downregulation of these genes may contribution to reduced hyphae formation and thus biofilm inhibition. TUP1 encodes a global transcriptional corepressor, repressing the expression of hypha-specific genes. A *tup1*Δ mutant displays hyper filamentation under conditions favorable for growth of the yeast form (Braun and Johnson, 1997, 2000). NRG1, which acts in concert with TUP1, encodes another transcriptional repressor. Hyper-filamentation is also observed upon the loss of either NRG1 or TUP1 (Braun et al., 2001). SK may inhibit filamentous growth of *C. albicans* by upregulating of TUP1 and NRG1. It should be noted that most of these genes can be regulated by farnesol. In our previous study, TUP1 was overexpressed in farnesol-treated *C. albicans* biofilm (Cao et al., 2005). Davis-Hanna et al. and other groups demonstrated the impact of Ras1-Cdc35-Efg1 signaling pathway as well as the downstream Hwp1 by farnesol (Davis-Hanna et al., 2008; Lindsay et al., 2012). Nevertheless, further studies are required to explore whether SK inhibits hyphae formation through enhancing farnesol production.

The formation of *C. albicans* biofilm includes three main stages: adhesion to biomaterial surfaces, growth to form an anchoring layer, and morphological transition to form a complex three-dimensional structure. CSH, which contributes to the interaction between the cells and the surfaces, is an important factor for cell adhesion (Luo and Samaranayake, 2002; Seneviratne et al., 2008). A positive correlation between biofilm formation and CSH has been reported in several studies (Samaranayake et al., 1995; Luo and Samaranayake, 2002; Pompilio et al., 2008). Previous studies revealed that CSH is correlated with cell adhesion, which is the first and important stage for biofilm formation (Luo and Samaranayake, 2002; Seneviratne et al., 2008). In this study, exposure of *C. albicans* biofilm to SK led to decreased CSH. Consistent with this, CSH1, which codes for a key CSH-associated protein, was downregulated upon SK treatment. In our previous study on the mechanisms of farnesol against *C. albicans* biofilm, farnesol could decrease CSH in a dose-dependent manner (Cao et al., 2005). Besides the downregulation of CSH1, the expression of ALS1 and ALS3, two ALS family genes that plays an essential role in promoting cell adhesion and biofilm formation (Sundstrom, 2002; Tronchin et al., 2008), were also decreased. These results implicated that SK might impede the initial adhesion of *C. albicans* cells, thus prevent biofilm formation.

Taken together, our study revealed the strong activity of SK against *C. albicans* biofilms and extended its potential application against biofilm-related fungal infection.

## Ethics Statement

The animal experiments were approved by the Animal Ethics Committee of the Second Military Medical University (Shanghai, China).

## Author Contributions

YC conceived and designed the experiments. YY, HM, FT, and HW performed the experiments. YY, FT, and YC analyzed the data. YC wrote the manuscript.

## Conflict of Interest Statement

The authors declare that the research was conducted in the absence of any commercial or financial relationships that could be construed as a potential conflict of interest.
